# Role of Artificial Intelligence in Surgical Training by Assessing GPT-4 and GPT-4o on the Japan Surgical Board Examination With Text-Only and Image-Accompanied Questions: Performance Evaluation Study

**DOI:** 10.2196/69313

**Published:** 2025-07-30

**Authors:** Hiroki Maruyama, Yoshitaka Toyama, Kentaro Takanami, Kei Takase, Takashi Kamei

**Affiliations:** 1 Department of Surgery, Tohoku University Graduate School of Medicine, Sendai, Japan; 2 Department of Diagnostic Radiology, Tohoku University Hospital, 1-1 Seiryo-Machi, Aoba-Ku, Sendai, Japan, Sendai, 980-8575, Japan, 81 227177312; 3 Department of Diagnostic Radiology, Tohoku University Graduate School of Medicine, Sendai, Japan

**Keywords:** LLM, ChatGPT, Japan Surgical Board Examination, surgical education, large language models, artificial intelligence, Medical Licensing Examination, diagnostic imaging

## Abstract

**Background:**

Artificial intelligence and large language models (LLMs)—particularly GPT-4 and GPT-4o—have demonstrated high correct-answer rates in medical examinations. GPT-4o has enhanced diagnostic capabilities, advanced image processing, and updated knowledge. Japanese surgeons face critical challenges, including a declining workforce, regional health care disparities, and work-hour-related challenges. Nonetheless, although LLMs could be beneficial in surgical education, no studies have yet assessed GPT-4o’s surgical knowledge or its performance in the field of surgery.

**Objective:**

This study aims to evaluate the potential of GPT-4 and GPT-4o in surgical education by using them to take the Japan Surgical Board Examination (JSBE), which includes both textual questions and medical images—such as surgical and computed tomography scans—to comprehensively assess their surgical knowledge.

**Methods:**

We used 297 multiple-choice questions from the 2021‐2023 JSBEs. The questions were in Japanese, and 104 of them included images. First, the GPT-4 and GPT-4o responses to only the textual questions were collected via OpenAI’s application programming interface to evaluate their correct-answer rate. Subsequently, the correct-answer rate of their responses to questions that included images was assessed by inputting both text and images.

**Results:**

The overall correct-answer rates of GPT-4o and GPT-4 for the text-only questions were 78% (231/297) and 55% (163/297), respectively, with GPT-4o outperforming GPT-4 by 23% (*P*=<.01). By contrast, there was no significant improvement in the correct-answer rate for questions that included images compared with the results for the text-only questions.

**Conclusions:**

GPT-4o outperformed GPT-4 on the JSBE. However, the results of the LLMs were lower than those of the examinees. Despite the capabilities of LLMs, image recognition remains a challenge for them, and their clinical application requires caution owing to the potential inaccuracy of their results.

## Introduction

Surgical training requires a considerable time commitment, as it includes various educational activities, on-the-job training, and supervised clinical experience [1]. In Japan, the surgery field is facing many challenges, such as the declining numbers of surgeons, regional health care disparities [2], and working-hour–related challenges [3]. Consequently, it is important to understand whether new technologies such as artificial intelligence (AI) and large language models (LLMs) can augment surgery education and training [4].

LLMs are AI systems trained on billions of words from papers, books, and other internet sources. ChatGPT—released by OpenAI in November 2022—is a generative AI chatbot that supports multimodal inputs and text generation, with a GPT as its backend [5]. ChatGPT has achieved conversational interactivity and human-like or better correct-answer rate across various fields—including the medical field [6]—suggesting that LLM applications could be beneficial in clinical, educational, and research settings [7].

GPT-4—released in March 2023—achieved an excellent correct-answer rate for United States Medical Licensing Examination (USMLE)-style questions, exceeding the passing threshold of 60% [8]. Moreover, in the field of surgery, GPT-3.5 obtained a 65% correct-answer rate for the US General Surgery Specialist Examination [9], and GPT-4 achieved a 76% correct-answer rate for the Korean Surgical Specialist Examination [10]. However, GPT-4 does not include an image-recognition function; consequently, questions that included images were excluded from both of these studies. To the best of our knowledge, no previous study has yet evaluated the correct-answer rate of LLMs on the Japan Surgical Board Examination (JSBE).

GPT-4-Vision (GPT-4V)—an improved version of GPT-4 with image-processing capabilities [6]—can process and interpret images along with text data, extending its potential application to areas that require image analysis. When both text- and image-based questions from the USMLE were input into GPT-4V, its correct-answer rate improved from 83.6% to 90.7% [11]. However, there have been no reports on the functional evaluation of AI in the field of surgery that includes image evaluations.

GPT-4 Omni (GPT-4o)—released in May 2024—features a considerably faster processing speed than GPT-4 and includes many upgrades, such as its improved non-English-language processing and enhanced visual and speech understanding [12]. Additionally, the GPT-4o knowledge base has been updated with data up to October 2023, enabling it to offer more accurate answers based on recent information and accurate text generation [13]. Several reports have evaluated the performance of Chat-GPT4o using medical examinations, but only 3 reports have evaluated the effectiveness of image input in addition to text [14-16]. Moreover, no study has yet evaluated it in the field of surgery. In many cases, diagnostic imaging plays an important role in surgical treatment plans, and specific images—such as intraoperative imaging findings—are sometimes used. Consequently, evaluating how LLMs handle surgery-specific images is critical for understanding their current capabilities. If LLMs have a high level of knowledge related to surgery-specific images, they have the potential to be effective tools in real clinical practice and surgical education.

There have been few reports evaluating the extent to which LLMs, such as GPT, possess surgical knowledge, particularly in relation to interpreting surgical images—a skill essential for clinical decision-making. This study aims to assess and compare the performance of GPT-4 and GPT-4o on JSBE, focusing not only on general surgical knowledge but also on image recognition and diagnostic accuracy. We examined the models’ responses to text-only and text-with-image questions using a retrospective evaluation design. We hypothesized that GPT-4o would outperform GPT-4, particularly on image-based questions. The findings of this study should be useful for medical educators and AI researchers seeking to understand the capabilities and limitations of LLMs in surgical education and training.

## Methods

### Question Dataset

This study used multiple-choice questions from the 2021‐2023 JSBE published by the Japan Surgical Society. Each question had five possible choices, with some requiring a single answer and others requiring two. The responses for the two-answer questions were deemed correct only if both correct answers were selected. The number of answers required was specified in the input text. Electronic versions of previous papers that were available for sale were also used. The Japan Surgical Society granted permission to answer these questions.

The JSBE is a multidisciplinary surgical knowledge examination designed for senior resident doctors in Japan who have completed a 3-year surgical training program. The number of examinees, successful candidates, and pass rates are listed in Table 1. There were 100 questions in each year, but 1 question in 2022 and 2 questions in 2023 were excluded as inappropriate questions, so a total of 297 questions were used in this study. The questions were presented in Japanese. To evaluate and compare responses to text-with-image questions, text-only questions were also included in the study. The text, obtained from an electronic question booklet, was entered into the models in an extensive markup language (XML) format. Moreover, screenshots of the test images were obtained from the booklet and saved in JPEG format, with their captions also being included.

**Table 1.** Annual test results of the Japan Surgical Board Examination.

Year	Examinees	Successful examinees	Pass rate (%)	Correct-answer rate (%)
2021	289	261	90.3	84.2
2022	1594	1534	96.2	92.7
2023	835	814	97.5	92.7

**Figure 1.**
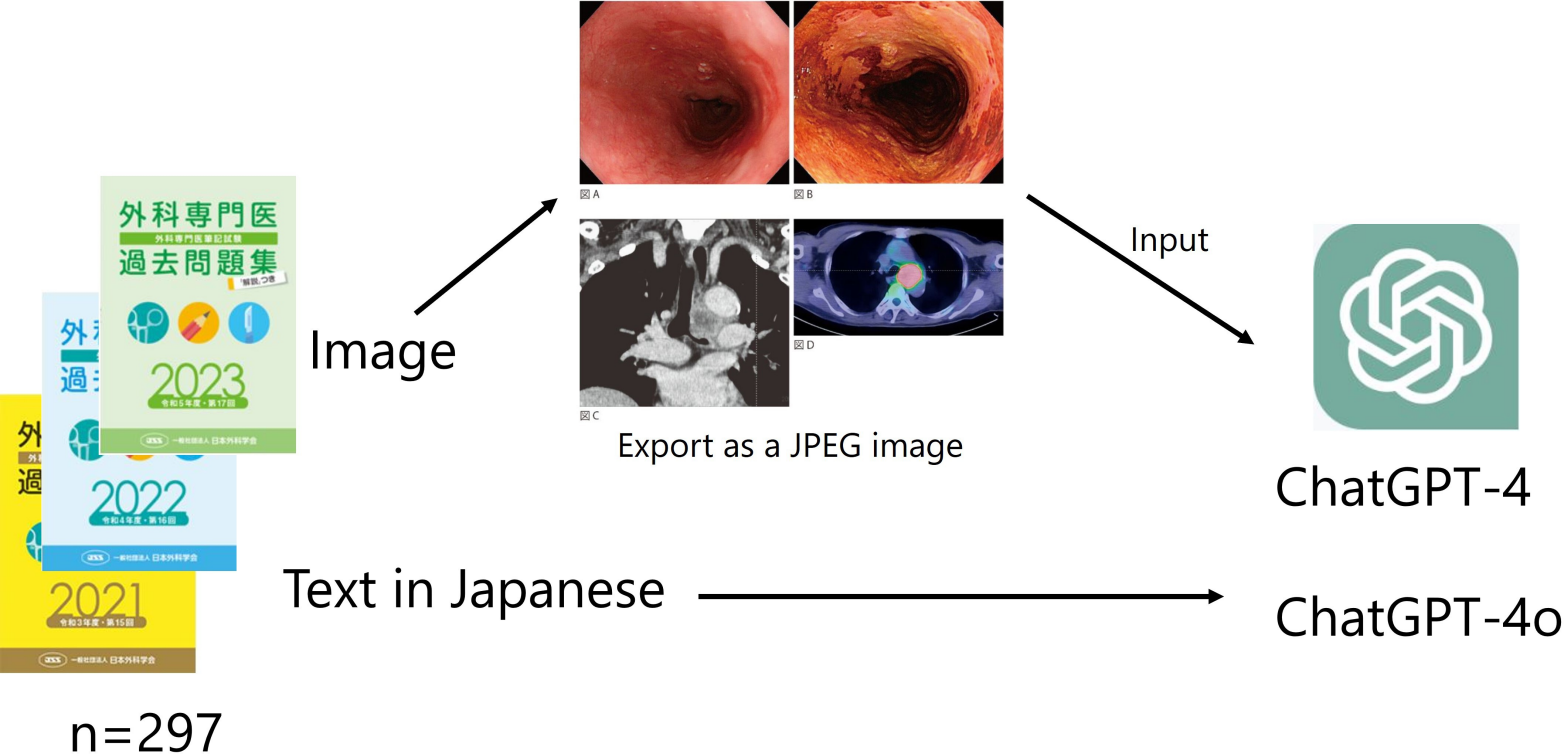
Collection of data from JSBE and input into GPT models. The questions were entered into an electronic booklet in Japanese. The images were saved as screenshots and input into ChatGPT-4o. JSBE: Japan Surgical Board Examination.

Questions with multiple images were exported and combined into a single image (Figure 1). The correct answers were also obtained from the electronic question booklet. The percentage of correct answers for each topic was calculated based on the number of correct answers provided by actual examinees for each question.

### Question Classification

The questions were classified based on whether they included images. Of the 297 questions, 104 included images (text-with-image questions), and the remaining 193 were text-only questions. They were grouped into 6 categories—that is, gastrointestinal surgery (134/297, 45.1%), cardiovascular surgery (44/297, 14.8%), thoracic surgery (30/297, 10.1%), pediatric surgery (30/297, 10.1%), breast and endocrine surgery (30/297, 10.1%), and emergency anesthesiology (29/297, 9.8%). The number of image modalities and images per question was as follows: the highest number of questions included computed tomography (CT) images (44/104, 42.3%), followed by endoscopy (15/104, 14.4%), and ultrasound (13/104, 12.5%) images. Additionally, X-ray (10/104, 9.6%), radiofluoroscopy (10/104, 9.6%), magnetic resonance imaging (MRI; 8/104, 7.7%), surface of skin findings (8/104, 7.7%), positron emission tomography (6/104, 5.8%), intraoperative findings (5/104, 4.8%), pathology (5/104, 4.8%), and other modality images (5/104, 4.8%) were also included. Furthermore, 44 out of 104 questions included 1 image (42.3%), 42/104 included 2 images (40.4%), 11/104 included 3 images (10.6%), 6/104 included 4 images (5.8%), and 1/104 included 6 images (which was the maximum; 1.0%). The percentage of correct answers for each topic was calculated based on the percentage of correct answers to the individual questions and was then compared with the percentages of correct answers provided by both GPT-4 and GPT-4o.

### Data Collection and Assessment

We used the GPT-4 and GPT-4o models via OpenAI’s application programming interface (API) without additional fine-tuning or custom configuration. All parameters were maintained at their default setting. No pretraining or fine-tuning was conducted, and no custom persona was provided. The questions were submitted via the OpenAI API in June 2024, and the GPT-4 and GPT-4o responses were collected. The internal GPT-4o and GPT-4 versions used in this study were gpt-4o-2024-05-13 and gpt-4-turbo-2024-04-09, respectively. GPT-4o was trained on data up to October 2023, whereas GPT-4 was trained on data up to December 2023 [17].

A maximum token limit of 4096 tokens was assumed, consistent with the default for many GPT-based API end points. All other parameters—for example, the temperature, top_p, and frequency penalty—were kept at their default settings.

All test questions were presented in Japanese. To ensure consistent model behavior and clear response formatting, the following English-language prompt was placed before each question: “Please answer the following question. Indicate the symbol of the option selected by you at the end.” This prompt was immediately followed by the question text in Japanese. This structure aimed to maintain response consistency across test items. The prompting strategy primarily followed a zero-shot format.

Questions with images were assessed twice—that is, once with and once without images. The answers that matched those in the question booklet were considered correct. Moreover, the percentage of correct answers was calculated for each image modality with the questions, and the percentage of correct answers was calculated based on the number of images included in the question. The percentage of correct answers for GPT-4o and GPT-4 were compared for all questions, text-only questions, and text-with-image questions, by the question category, image modality used, and number of images. For category-by-category comparisons, only the results for questions with image inputs were compared, but for the other items, the results with and without image inputs were also compared.

### Statistical Analyses

McNemar test was used to compare the proportion of correct responses between the GPT-4 and GPT-4o. Fisher exact test was used for each category—that is, with or without images, lower-order thinking versus higher-order thinking, and 2 answers versus 1 answer—to assess the GPT-4o correct-answer rate for each category. Additionally, a chi-square test was conducted to compare grades across topics. All tests were 2-tailed, and *P* values<.05 were considered significant. All *P* values were nominal and were not corrected for multiple comparisons. The statistical analyses were conducted using JMP Pro 17.0 (SAS Institute Inc).

### Ethical Considerations

This study did not include human participants or patient data. All the data used in this study are publicly available. Therefore, it was excluded from review by the Institutional Review Board of Tohoku University (IRB number 11000629).

## Results

### Correct-Answer Rates of GPT-4 and GPT-4o

Of the 297 questions used for the text-input only test, GPT-4 answered 164 (55%) correctly and GPT-4o answered 225 (76%) correctly; thus, GPT-4o outperformed GPT-4 by 21% (*P*<.001). Additionally, when image inputs were performed for the 104 text-with-image questions out of the 297 questions, GPT-4o outperformed GPT-4 by 23% (*P*<.001), providing 231 (78%) and 163 (55%) correct answers, respectively. Comparisons of their correct-answer rates for individual groups showed that GPT-4o provided significantly more correct answers for image-based questions (GPT-4o 67% vs GPT-4 45%; *P*=<.002), text-only questions (83% vs 61%; *P*=<.001), digestive surgery (70% vs 42%; *P*=<.001), cardiovascular surgery (98% vs 68%; *P*<.00031), and breast and endocrine surgery (93% vs 67%; *P*=.0047). However, no significant differences (GPT-4o vs GPT-4) were evident between their correct-answer rates for questions related to thoracic surgery (63% vs 57%; *P*=.41), emergency surgery and anesthesia (86% vs 66%; *P*=.06), and pediatric surgery (73% vs 70%; *P*=.71). Notably, GPT-4o provided more correct responses than GPT-4 (Table 2).

**Table 2.** GPT-4 and GPT-4o correct-answer rates for the Japan Surgical Board Examination.^a^

Question type	Number of questions	Image input	Correct-answer rate		*P* value
			GPT-4, n (%)	GPT-4o, n (%)	
All questions	297	–	164 (55)	225 (76)	.001
		+	163 (55)	231 (78)	.001
Text-with-image questions	104	–	46 (44)	64 (62)	.002
		+	47 (45)	70 (67)	.<001
Text-only questions	193	–	118 (61)	161 (83)	.<001
Topic					
Digestive surgery	134	+	56 (42)	94 (70)	.<001
Cardiovascular surgery	44	+	30 (68)	43 (98)	.<001
Thoracic surgery	30	+	17 (57)	19 (63)	.41
Pediatric surgery	30	+	21 (70)	22 (73)	.71
Breast and endocrine surgery	30	+	20 (67)	28 (93)	.005
Emergency and anesthesia	29	+	19 (66)	25 (86)	.06

a Data are presented as the number of correct answers.

### GPT-4o’s Correct-Answer Rate on Text-With-Image Questions Compared With its Rate on Text-Only Questions

Even when image inputs were used for the text-with-image questions, GPT-4o provided 67% correct answers, compared to 83% for the text-only questions, indicating a statistically significant difference (*P*<.002).

### Correct-Answer Rate With and Without Image Input

The percentages of correct responses provided by both models with and without image inputs were compared for 104 text-with-image questions—here, GPT-4o provided correct-answer rates of 62% and 67% with and without image inputs (*P*=.2), respectively, whereas GPT-4 provided correct-answer rates of 44% and 45% with and without image inputs (*P*=.86; Table 3).

**Table 3.** GPT-4 and GPT-4o correct-answer rates based on image-input and no image-input questions.^a^

Large language model	Input image, n (%)^b^	No input image, n (%)^b^	*P* value
GPT-4	47 (45)	46 (44)	.86
GPT-4o	70 (67)	64 (62)	.20

a Data are presented as the number of correct answers. Values in parentheses indicate the percentage of correct responses.

b The percentage indicates the percentage of correct answers to the 104 text-with-image questions.

### Correct-Answer Rate Comparison of GPT-4 and GPT-4o by Category

GPT-4 provided the highest percentage of correct answers for pediatric-surgery questions (70%) and the lowest for gastrointestinal-surgery questions (19%; *P*=.0027). By contrast, GPT-4o provided the highest percentage of correct answers for cardiovascular-surgery questions (98%) and the lowest for thoracic-surgery questions (63%; *P*=.002). The correct-answer rate for the examinees referred to here is the correct-answer rate for all examinees from 2021 to 2023 (Table 4).

**Table 4.** GPT-4o, GPT-4, and examinees’ correct-answer rates across various categories.

Topic	Number of questions	Text-with-image questions, n (%)	Correct-answer rate (%)		
			GPT-4	GPT-4o	Examinees
All questions	297	104 (35)	55	78	90
Digestive surgery	134	43 (32)	42	70	89
Cardiovascular surgery	44	19 (43)	68	98	91
Thoracic surgery	30	17 (57)	57	63	88
Pediatric surgery	30	11 (37)	70	73	92
Breast and endocrine surgery	30	7 (23)	67	93	90
Emergency and anesthesia	29	7 (24)	66	86	91
*P* value	—^a^	—^a^	.003	.<001	

a Not applicable.

### Comparison of GPT-4 and GPT-4o Responses by Image Modality and Number of Figures

Using the text-with-image questions, the correct-answer rates for GPT-4 and GPT-4o were compared using various imaging modalities and images. GPT-4 provided the highest percentage of correct answers for questions on radiofluoroscopy and inspection (70% and 75%, respectively), whereas GPT-4o provided the highest percentage of correct answers for radiofluoroscopy and ultrasound (80% and 92%, respectively). By contrast, the correct-answer rates of the models were low for questions that included intraoperative and pathological findings—that is, they were 20% and 40% for GPT-4, respectively, and 40% for GPT-4o for both intraoperative and pathological findings. Moreover, a weak negative correlation was evident between the number of images and the percentage of correct answers, but it was not statistically significant (Table 5).

**Table 5.** Correct-answer rate comparisons based on imaging modality and number of images.^a^

Variables	n	Correct-answer rate, n (%)			
		GPT-4		GPT-4o	
		Image input +	Image input −	Image input +	Image input −
Imaging modality					
Text-with-image questions	104	47 (45)	46 (44)	70 (67)	64 (62)
XP^b^	10	5 (50)	4 (40)	6 (60)	5 (50)
Radiofluoroscopy	10	7 (70)	6 (60)	8 (80)	6 (60)
Ultrasound	13	6 (46)	8 (62)	12 (92)	10 (77)
CT^c^	60	25 (42)	27 (45)	39 (65)	37 (62)
MRI^d^	8	5 (63)	2 (25)	5 (63)	4 (50)
PET^e^	6	4 (67)	2 (33)	3 (50)	2 (33)
Endoscopy	15	9 (60)	8 (53)	9 (60)	11 (73)
Surface of skin findings	8	6 (75)	4 (50)	5 (63)	6 (75)
Intraoperative findings	5	1 (20)	2 (40)	2 (40)	3 (60)
Pathology	5	2 (40)	2 (40)	2 (40)	2 (40)
Other	5	2 (40)	3 (60)	5 (100)	4 (80)
Number of figures					
1	44	19 (43)	18 (41)	28 (64)	26 (59)
2	42	18 (43)	19 (45)	32 (76)	27 (64)
3	11	7 (64)	8 (73)	10 (91)	9 (82)
4	6	3 (50)	1 (17)	1 (17)	1 (17)
6	1	0 (0)	0 (0)	0 (0)	0 (0)

a Data are presented as the number of correct answers. Values in parentheses indicate the percentage of correct responses.

b XP: X-ray photograph.

c CT: computed tomography.

d MRI: magnetic resonance imaging.

e PET: positron emission tomography.

## Discussion

### Principal Findings

GPT-4o significantly outperformed GPT-4 across all evaluated categories. However, neither GPT-4 nor GPT-4o achieved examinee-level accuracy for any question (Table 1). The correct-answer rates for text-only questions were higher than those for text-with-image questions for both models. Moreover, the inclusion of image inputs did not lead to a significant improvement in performance on text-with-image questions (Table 3). Performance varied by image type, with particularly low correct-answer rates for questions involving intraoperative and pathological images. By contrast, the correct-answer rates were relatively higher for radiological images such as CT and MRI images.

The results showed that there was no significant difference in the percentage of correct responses between GPT-4 and GPT-4o for thoracic surgery, emergency and anesthesia, and pediatric surgery. When comparing GPT-4o to the results of examinees, both demonstrated similarly low correct-answer rates for questions related to thoracic and gastrointestinal surgery. There was no consistent pattern evident in terms of which surgical category exhibited the highest correct response rate.

### Additional Analysis by Problem Type

In terms of category-specific differences, the percentage of correct responses for both GPT-4 and GPT-4o did not differ significantly for thoracic, emergency and anesthesia, and pediatric surgery. A comparison of GPT-4o and examinee correct-answer rates demonstrated similarly low correct response rates for questions related to the thoracic and gastrointestinal surgeries. However, no consistent trend was evident with the highest percentage of correct responses. The correct-answer rate was low for thoracic surgery because questions in this field comprised a high proportion of text-with-image questions. By contrast, it was high for breast and endocrine surgery and emergency and anesthesiology, which comprised fewer text-with-image questions. However, the opposite trend was evident for cardiovascular surgery, where no consistent trend was evident in the correct-answer rates for text-with-image questions. This result could be attributed to the fact that many of the questions could be answered correctly without image recognition, or that many of the images were easy to understand even when image recognition was required.

### Responses to Intraoperative Imaging Problems

An additional study on the correct-answer rate was conducted to assess the differences in the GPT model responses based on the imaging modality. This is only a hypothesis, but owing to the small sample size, as 1 question contained several types of images, the correct-answer rate was more than 20% lower than the overall GPT-4 correct-answer rates for questions involving intraoperative findings and those of GPT-4o for intraoperative and pathological images compared with those for radiological modalities such as CT and MRI (Table 4). Only questions involving intraoperative findings had a correct-answer rate after image input that was more than 20% lower than the average for both GPT-4 and GPT-4o, which could be considered to be a GPT image-recognition weakness. Additionally, the responses to intraoperative images were evaluated individually (Multimedia Appendix 1). Although liver resection was identified in intraoperative liver-resection images, the actual resection was misidentified. Moreover, in images of mediastinal tumors, the tumor and recurrent nerve were either not mentioned or could not be identified, whereas from the intraoperative inguinal-hernia images, the arteriovenous vein in the inferior abdominal wall was misidentified as the vas deferens.

### Implications of Findings

From this research, it is evident that GPT-4o significantly outperformed GPT-4 across all evaluated categories, indicating that OpenAI’s model development is progressing steadily. However, despite these improvements, neither GPT-4 nor GPT-4o achieved the correct-answer rates of actual examinees. This highlights that current LLMs, while advancing rapidly, still fall short of the reliability required for high-stakes clinical decision-making or licensing-level assessments. In particular, GPT-4o exhibited lower accuracy on text-with-image questions compared to text-only questions, and the inclusion of image inputs did not significantly improve its performance. This reflects an ongoing limitation in the image recognition capabilities of LLMs, especially for complex visuals such as intraoperative and pathological images, and suggests that caution is warranted when considering these models for clinical use.

However, this study was conducted without pre-tuning, and the accuracy of LLMs could be potentially improved by tuning them in a field-specific manner [7]. Pretuning them on data from medical textbooks and previous examinations could enhance the relevance and accuracy of their responses. Pretraining has the potential to improve model performance, but the process can be complicated and is not supported by some models. The results of the models that did not undergo pretraining can be said to be results that can be applied to general readers and various models.

A “Socratic tutor mode” educational application of GPT-4o has been reported, wherein the complexity of medical questions can be changed during the conversation based on the learner’s understanding [7]. In this study, GPT-4o provided a high percentage of correct answers to text-only questions, which could be used for learning guideline content and obtaining general surgical knowledge, where the answers are clear to residents and majors studying surgery. Additionally, if its image-recognition capabilities improve in the future and it becomes possible to diagnose intraoperative images specific to surgery with a high degree of confidence, it could become a useful indicator when making decisions in daily clinical practice.

### Comparison to the Literature

Previous studies have shown that the GPT-4 correct-answer rate could be improved for USMLE by using images to complement the text input [11]. However, similar to our study, researchers have reported that inputting image information did not increase the percentage of correct answers [16,18-22]. Additionally, a previous study has suggested that GPT-4 prioritizes verbal information over images [20].

Previous reports [23] have shown that ChatGPT is able to provide more accurate responses when given English-language input than non-English-language input, but it has also been shown that the correct-answer rate of GPT-4o has improved when given Japanese-language inputs [12]. Moreover, the results of this study reflect this fact, which is consistent with the findings of a previous study on radiology [14], highlighting GPT-4o’s enhanced reasoning and better responsiveness to Japanese inputs, thereby successfully addressing the limitations of earlier variants.

### Strengths and Limitations

It should be noted that, unlike previous studies which relied on recalled questions or researcher-derived answers [9,10], commercially available past examinations were used in this study to ensure a more accurate and reliable assessment, making it a strong point of our research. Nonetheless, this study had several limitations. First, the LLMs were only asked each question once; however, LLMs are generative models, often referred to as “probabilistic parroting” models [24]. This is because they generate answers based on the probability of selecting the most appropriate word from the training data. Consequently, different answers can be returned for the same question with a certain probability when asked multiple times [25]. To address this problem, it is necessary to ask the same question multiple times and assess the degree to which the answers fluctuate. Second, ChatGPT responses can be interspersed with answers based on false evidence or factual errors, commonly referred to as “hallucinations,” which is the phenomenon of asserting incorrect content as if it were correct [26,27]. Even though such responses can be determined to be false by specialists, they can confuse doctors during training. This phenomenon occurs even as the correct-answer rate of the model improves—that is, the greater the confidence in responses, the more difficult it can become to identify incorrect information. Third, LLMs—including ChatGPT—are updated periodically, which could alter their correct-answer rate or incur unexpected pretraining as the questions are entered. Consequently, the reproducibility of test results in future studies remains uncertain. Finally, we used a relatively small number of questions, which could have resulted in an inadequate analysis, particularly for the category-specific correct-answer rate. The differences in the correct-answer rate between GPT-4o and GPT-4 were substantial in the fields of cardiovascular surgery, digestive surgery, and breast and endocrine surgery; however, differences in other fields were minimal, which could be attributed to the limited sample size. If the JSBE is able to obtain more high-quality problems as it continues to hold more events, it could be possible to evaluate models with an even greater correct-answer rate.

In conclusion, GPT-4o outperformed GPT-4 for the JSBE. Although there is still room for improvement in image recognition and clinical applications, which should be approached with caution, the results suggest that improved models and pretraining could provide LLMs with more accurate medical knowledge and enhance their clinical judgment, which could be useful in enhanced learning for surgeons.

## Supplementary material

10.2196/69313 Multimedia Appendix 1 LLM's response to intraoperative findings.

## Abbreviations

AI: artificial intelligence
API: application programming interface
CT: computed tomography
JSBE: Japan Surgical Board Examination
LLM: large language model
MRI: magnetic resonance imaging
USMLE: United States Medical Licensing Examination
