# Supplementary material for: Role of Artificial Intelligence in Surgical Training by Assessing GPT-4 and GPT-4o on the Japan Surgical Board Examination With Text-Only and Image-Accompanied Questions: Performance Evaluation Study
Source: JMIR Med Educ. 2025 Jul 30;11:e69313. doi: 10.2196/69313 (PMC12310146; doi:10.2196/69313)
Supplement: Multimedia Appendix 1 [file mededu-v11-e69313-s001.docx]

**Supplementary Table 1** Responses of GPT-4 and GPT-4o to questions with intraoperative images

|  | Question A | Question B | Question C |
| --- | --- | --- | --- |
| Intraoperative findings | Intraoperative findings of hepatectomy | Thoracoscopic findings of upper mediastinal tumors | Laparoscopic findings in the inguinal region |
| Matters to be diagnosed | Liver resection techniques | Possible complications during tumor resection | Anatomy in the inguinal region |
| Correct answer | Resection of the left and caudate lobes of the liver, bile duct resection | Recurrent nerve paralysis | Inferior abdominal wall artery |
| GPT-4 Answer | Resection of the right and caudate lobes of the liver, bile duct resection | Recurrent nerve paralysis | Seminal duct |
| Assessment of image by GPT-4 | Site of the resected liver is misjudged. | No reference to images | Point out that that is seminal duct |
| GPT-4o answer | Resection of the right and caudate lobes of the liver, bile duct resection | Recurrent nerve paralysis | Seminal duct |
| Assessment of image by GPT-4o | Hepatic resection and bile duct resection are mentioned, but the site is not noted. | Mentions the operative field of the anterior mediastinum and the presence of an aortic arch/tumor. | Point out that that is seminal duct |
